# Exploring the Acute Effects of Immersive Virtual Reality Biking on Self-Efficacy and Attention of Individuals in the Treatment of Substance Use Disorders: A Feasibility Study

**DOI:** 10.3390/brainsci14070724

**Published:** 2024-07-18

**Authors:** Evlalia Touloudi, Mary Hassandra, Vasileios T. Stavrou, Fotis Panagiotounis, Evangelos Galanis, Marios Goudas, Yannis Theodorakis

**Affiliations:** 1 Department of Physical Education and Sport Sciences, University of Thessaly, 42100 Trikala, Greece; elina.touloudi1995@gmail.com (E.T.); mxasad@uth.gr (M.H.); egalanis@uth.gr (E.G.); mgoudas@pe.uth.gr (M.G.); 2 Laboratory of Cardio-Pulmonary Testing and Pulmonary Rehabilitation, Respiratory Medicine Department, Faculty of Medicine, University of Thessaly, 41110 Larissa, Greece; vasileiosstavrou@hotmail.com; 3 Department of Education, KETHEA, 11636 Athens, Greece; panagiotounisfotis@gmail.com

**Keywords:** virtual reality, exercise, substance use disorders, self-efficacy, attention

## Abstract

Background: The role of exercise programs during substance use disorder (SUD) treatment is considered particularly supportive in fostering positive psychological and behavioral changes. The treatment of SUD is a challenging and demanding process. Therefore, it is crucial to discover innovative methods to enhance and facilitate it. Integrating exercise into a virtual environment is an innovative approach for drug rehabilitation, offering multiple benefits to individuals undergoing treatment. The aim of this feasibility study was to explore the acute effects of cycling exercise in an immersive virtual reality (VR) environment on attentional control and self-efficacy expectations of individuals undergoing SUD treatment. Methods: A total of 20 individuals (mean age of 37.75 years) enrolled in a SUD treatment program participated in the study. They were instructed to complete a single session of cycling with a self-selected duration within the VR system. Results: Following the cycling session, pre-post measures indicated a statistically significant improvement in self-efficacy expectations and attentional control measured with the Stroop test. The results revealed that participants expressed high levels of intention for future use, interest/enjoyment, and positive attitudes toward the VR exercise system. Qualitative data indicated that participants found the VR exercise system enjoyable, expressed a strong intention to use it, and did not encounter technical difficulties or negative emotions. Conclusions: In conclusion, it seems that engaging in a brief period of self-selected exercise within a virtual environment can result in temporary activation of cognitive changes, heightened self-efficacy expectations, and a motivating approach to increasing physical activity participation among individuals with SUD, thus suggesting the feasibility of this approach.

## 1. Introduction

Drug use represents a major issue for public health, with potential consequences including substance use disorders (SUD), mental health disorders, HIV infection, cancer, cirrhosis of the liver, hepatitis, overdose, premature death, and impaired cognitive function with chronic use [1]. In general, addiction is a disorder that affects how the brain processes information. The areas of the brain impacted by SUD are the same ones involved in important cognitive functions like learning, memory, attention, reasoning, and controlling impulses. Chronic drug use can alter brain structure and function, leading to addiction [2] and affecting memory and cognitive functions [3]. Drugs have negative effects on natural cognitive function, promote drug use, and hinder the development of behaviors supporting abstinence. During substance abstinence, individuals may experience cognition-related withdrawal symptoms caused by many drugs that are usually temporary, in contrast with long-term use symptoms that lead to lasting cognitive declines. Moreover, cognitive deficits related to drugs may have negative effects on the individual’s well-being [4].

Individuals undergoing SUD treatment experience notable physical and cognitive benefits when incorporating exercise into their regimen. Research suggests that physical activity positively influences attention [5], contributing to enhanced cognitive functioning and potentially supporting addiction recovery. Exercise programs and physical activities appear to effectively reduce substance cravings and support abstinence. They serve as beneficial elements for both prevention and intervention, enhancing the impact of traditional therapy. Some of the benefits of exercise are prevention of relapse, repair of cardiovascular and muscular damage, reduced symptoms of anxiety and depression and improved mood, increased self-confidence, self-esteem, and body image, improved well-being and quality of life, personal satisfaction, optimism, and adopting a healthy lifestyle [6,7,8]. Systematic reviews have highlighted the critical role of physical activity and exercise programs in the treatment of SUD [9,10].

Cognitive functions are significantly impaired by substance use, leading to reduced memory, attention, and executive functioning [11]. These cognitive deficits are a critical risk factor for treatment dropouts [12]. Attention is a large and complex topic involving psychology and neuroscience. Generally, attention could be described as the flexible control of limited computational resources [13]. Many researchers have studied the influence of acute exercise on the Stroop effect (used widely to assess attention deficits) in healthy and clinical populations. The findings of a recent study, in which the Stroop test was applied pre, post, and 40-min post-resistance exercise, supported that acute exercise can lead to improved scores [14]. The use of a virtual reality platform can also offer cognitive flexibility and selective attention improvements in young males, as exercise does offer such benefits [15]. Therefore, a combination of VR and exercise may contribute to cognitive development. Furthermore, current investigations are delving into the potential advantages of high-intensity interval training for enhancing cognitive function in individuals with SUD [16]. Additionally, a recent randomized controlled trial revealed significant enhancements in cognitive functions and emotional well-being among patients with SUD engaging in a group aerobic exercise program [17]. Notably, these findings underscore the promising role of exercise interventions in addressing cognitive deficits associated with SUD.

Self-efficacy is defined as the expectation that one can successfully perform a specific behavior required to produce a certain outcome [18]. Positive self-efficacy is related to individuals’ perceptions of their capability to control their own functioning and the events that affect their lives while decreased self-efficacy may lead to doubt and easily giving up in the face of obstacles [19,20]. Research by Schwarzer et al. 2014 [21] indicates a strong connection between self-efficacy and both physical and mental health. There are, also, studies supporting that the consumption of illicit drugs is likely to impair individuals’ self-control and self-efficacy [22]. Additionally, other studies support the low levels of self-efficacy during the treatment process [23]. According to Kadden and Litt (2011) [22], self-efficacy plays a crucial role in the treatment SUD and significantly influences treatment outcomes. Therefore, it is essential to identify effective strategies to enhance self-efficacy in individuals undergoing this process. In accordance with that, other researchers support that there is a significant positive correlation between self-efficacy and treatment motivation [24]. Higher levels of self-efficacy, quality of life, and social support were found to be inversely related to the likelihood of relapse in individuals with drug addiction [25,26]. The role of attitudes and intentions toward participation in sports and exercise programs has been documented. Positive attitudes and intentions towards substance abuse treatment contribute to higher rates of completing the treatment program. Attitude and control components are positively associated with intention and treatment participation or completion [19,20]. Furthermore, engaging in exercise during drug addiction therapy can enhance physical fitness outcomes and positively impact psychological well-being, fostering improvements in personality traits and psychological aspects such as self-confidence. Finally, exercise-induced improvements in self-efficacy can contribute to overall well-being and quality of life, promoting a positive mindset and sense of achievement throughout the recovery journey [27].

Self-efficacy expectations, as outlined by Bandura (1977) [18], pertain to an individual’s belief in their capability to successfully execute behaviors required to produce specific outcomes in particular situations. This concept emphasizes the confidence an individual has in their ability to achieve desired results through their actions within specific contexts. The distinction between self-efficacy and self-efficacy expectations lies in their scope and focus. Self-efficacy encompasses a broader sense of overall competence and belief in one’s abilities across various tasks and situations. In contrast, self-efficacy expectations are more specific, relating to an individual’s confidence in their capacity to perform particular behaviors to attain specific outcomes in defined circumstances [18].

Virtual reality (VR) is a computer-generated simulation of a real or imaginary three-dimensional environment that allows users to explore and interact with it. In recent decades, VR has been increasingly used not only for entertainment and leisure but also for exercise, educational purposes, rehabilitation, health, and other applications [28]. Virtual environments can simulate reality without limitations in space and time, which makes them more attractive [29]. Exercise in a VR environment has been a rising method of exercise in recent years. Several studies were involved with VR exercise and support that it is suitable to offer beneficial effects on physiological, psychological, and rehabilitation factors compared to traditional exercise methods [30]. It has also been shown that VR exercise has favorable effects on cognitive factors such as functional ability, attention, and memory and mental health conditions like anxiety and depression [31]. Exercise in VR environment combined with cognitive tasks, according to another study, appeared to be appropriate and had positive effects even in people with mild cognitive impairment [32]. Additionally, recent research by Krommidas et al. (2022) [33] has highlighted the potential of acute exercise and VR tasks to positively impact children’s memory function and exercise preference, providing valuable insights into the broader applications of VR-based exercise interventions. VR environments are also a reliable way for providing information about addictive disorders, such as the desire to use, and can influence emotional state, attention, and cognitive function. Individuals with attention deficits, or those who face difficulties participating in group activities, may benefit from the virtual environment [34].

To the best of our knowledge, there are few studies in the literature evaluating the effect of exercise in a VR environment in populations in the treatment of SUD. According to what was mentioned above, it would be important to explore whether exercise in a VR environment could help to improve the self-efficacy expectations and attentional control of individuals in drug addiction treatment and be a useful and attractive tool to enhance their participation in an exercise program.

### Research Questions and Hypotheses

In the present study, we sought to address several key research questions related to the utilization of VR exercise as an intervention for individuals undergoing SUD treatment. Firstly, we investigated whether engaging in exercise with the VR environment would lead to a significant improvement in self-efficacy expectations among participants in the SUD treatment program. We hypothesized that participation in VR exercise would be associated with a noteworthy increase in self-efficacy expectations. Additionally, we explored potential differences in attentional control levels, as measured by the Stroop test, before and after a single session of cycling using the VR system. We hypothesized that this intervention will decrease Stroop reaction time in people undergoing SUD treatment. Lastly, we delved into participants’ attitudes, intentions for future use, and levels of interest/enjoyment regarding exercise within the VR system. We hypothesized that individuals in the SUD treatment program would exhibit positive attitudes, express strong intentions for future use, and report high levels of interest/enjoyment concerning VR-based exercise. Overall, these research questions and hypotheses were designed to comprehensively investigate the potential benefits of VR exercise within the context of SUD treatment.

Therefore, the aim was to explore the acute effects of pre- and post-exercise in a VR environment on self-efficacy expectations and attentional control of individuals in SUD treatment. Additionally, their attitudes, intentions for future use, and interest/enjoyment about this type of exercise were investigated.

## 2. Materials and Methods

### 2.1. Participants & Setting

A convenience sample of 20 individuals in SUD treatment of KETHEA ITHAKI therapeutic community participated. KETHEA ITHAKI is a therapeutic community located in Sindos, Thessaloniki, Greece, that aims to help individuals struggling with addiction and substance abuse with a residential program without substitution (https://www.kethea.gr, accessed on 12 May 2024). The inclusion criteria encompassed individuals between the ages of 18 and 60, who were functionable and capable of participating in exercise, without colorblindness, and without significant psychiatric conditions including severe anxiety disorder, psychosis, aggressive and violent behavior, psychopathic, schizophrenic, or paranoid personality disorders, or hallucinations. Screening based on the inclusion criteria for participants in the study was conducted by the staff of KETHEA, where the study took place. It is important to mention that most participants had multiple unsuccessful attempts at SUD treatment before the study was conducted. Table 1 presents the days they were in treatment during their latest attempt. According to an article from KETHEA (Therapy Center for Dependent Individuals) published in 2017, the ratio of women to men participating in SUD treatment programs is approximately 1 to 5. This indicates that approximately 20% of all participants in the program are women. (https://www.kethea.gr/nea/oie-anagki-gia-ypostiriksi-ton-eksartimenon-gynaikon/, accessed on 12 May 2024). Their demographic characteristics are presented in Table 1.

### 2.2. Study Design

The study employed a quasi-experimental research design, chosen for its appropriateness in the context of being both exploratory and constrained by practical considerations. The rationale for adopting this design stems from the exploratory nature of the research, as only a limited number of studies have utilized VR exercise for this particular population. Furthermore, the feasibility of alternative designs was restricted by the fact that the therapeutic community KETHEA ITHAKI had only 20 participants. Additionally, ethical and practical concerns were highlighted, as raised by the SUD therapists. They believed that it would be considered unethical or problematic to expose only a limited number of participants to the proposed VR exercise experience.

### 2.3. Ethics Approval and Consent to Participate

The studies involving human participants received approval from the institution’s ethics committee (approval number: 1829, 13 October 2021). Personal data confidentiality is guaranteed under regulation (EU) 2016/679 (General Data Protection Regulation). Everyone involved in KETHEA, including participants and medical staff, received verbal explanations of the procedure. Participants were given written information and signed consent forms. They were also encouraged to ask questions and received thorough responses.

### 2.4. VR System

In our study, we employed a mixed methods approach, integrating both quantitative and qualitative methodologies. This comprehensive strategy allowed us to benefit from the strengths of each method, enhancing the depth and completeness of our findings. The methodology followed was based on our previous studies examining the effects of the VR exercise system on patients with mild cognitive impairment and female office workers [32,35].

The VR system consists of a cycle-ergometer connected to a computer, the VR head-mounted display and controllers, and the VR application. There is a detailed description of the equipment in our previous study [35].

**Figure 1:**
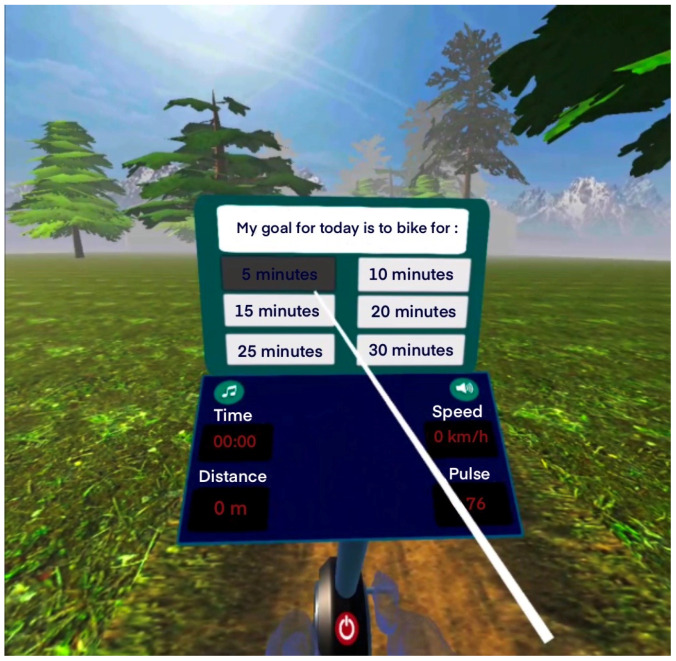
Selection of exercise duration in the VR system [35].

The VR application allows users to set their exercise goals (such as duration) (Figure 1), receive feedback on their training performance (including cycling duration and total distance), customize tasks (such as selecting music), and monitor their progress (displaying distance, time, and speed data on screen). Additionally, users could select their preferred virtual landscape (forest, beach, snowy terrain) and choose motivational words or phrases (like “calmly”, “I can”, “I will do well”, “Very nice”, or option for no verbal cues). The exercise duration was determined beforehand by the participants. In general, participants were allowed to choose the duration, landscape, motivational words or phrases, and music to enhance their experience and motivation. A VR controller with ray casting serves as the selection tool, allowing users to interact by pointing the ray at buttons and pressing the trigger button.

**Figure 2:**
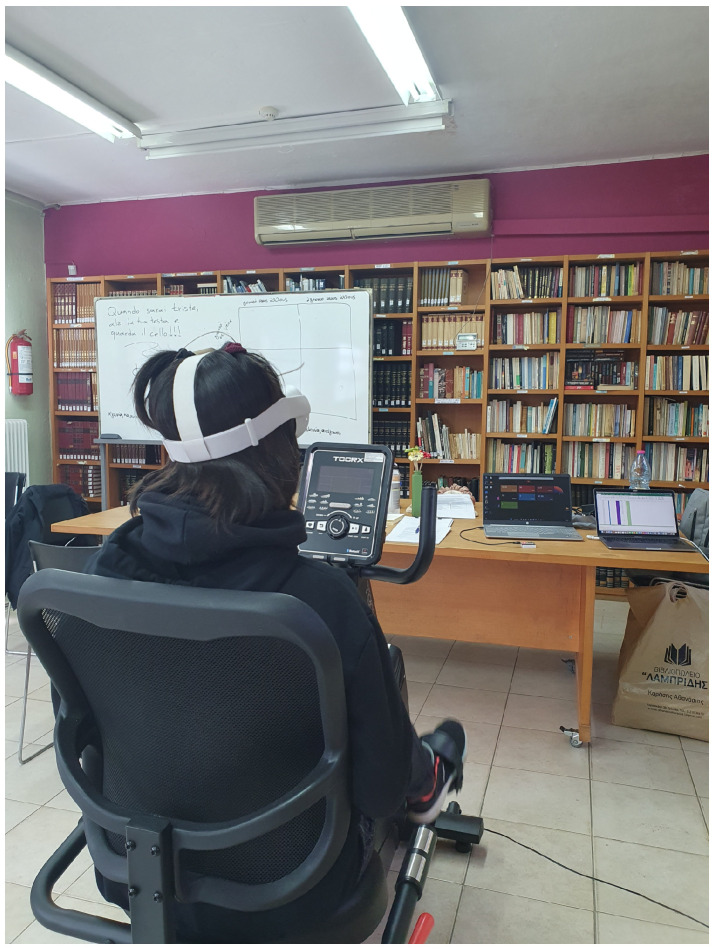
Participant cycling in the VR environment.

After that, each participant starts the cycling performance (Figure 2), and at the end, they receive feedback from the application about the time and distance they achieved and they can assess their experience through structured evaluation questions., e.g., “Are you tired today?”, “Did you like the way you exercised today?”, “How often did you repeat the word or phrase during the exercise?”

The VR training system employed is named VRADA (VR Exercise App for Dementia and Alzheimer’s Patients), Version 4.4. This VR system (Version 3.7) was utilized with university students and patients experiencing mild cognitive impairment. It functioned as a dual-task training tool designed to enhance both physical and cognitive health. The study aimed to evaluate its acceptance, tolerance, and usability among participants [32]. Additionally, Baldimtsi et al., 2023 [36] utilized the same VR system with older adults diagnosed with MCI, conducting 32 sessions focusing on both physical and cognitive training. Their findings suggest that the VR system effectively improves cognitive abilities. Given the promising results from both studies, further investigation into the applicability of VRADA across diverse populations could be warranted. The VRADA system, originally intended for dementia or Alzheimer’s patients, underwent modification in our study. We utilized an updated version excluding cognitive tasks during exercise, opting for questionnaires more suitable for this population, focusing on aspects such as attention and self-efficacy expectations.

The VRADA system offers benefits that cater to the needs of individuals in the treatment of SUD, such as mood improvement, anxiety, and depression reduction. According to the results of a study, that examined the impacts of acute VR exercise paired with cognitive activities on children’s memory function and their preference for exercise, children who experienced VR exercise noted higher scores in memory tests compared with children who experienced a traditional cycling session and control group. Additionally, children showed high levels of enjoyment, intention for future use, and attitudes toward cycling in a VR environment [33]. Acute VR exergaming can also lead to mood improvement according to Ochi et al. (2022) [37]. Furthermore, it serves as an enjoyable and appealing method of exercise that could potentially benefit individuals struggling with addiction by promoting physical health and mitigating adverse effects of treatment factors such as withdrawal symptoms, relapse, treatment burnout, etc.

### 2.5. Procedure

All participants were briefed on the purpose of this study and completed the consent form. The experimental procedure was completed in one visit and based on our previous study [34]. Then, they filled in a questionnaire with questions about their demographic characteristics, the TCU Drug screening questionnaire [38] and the IPAQ [39].

After that, each participant adjusted their seat on the stationary bike and familiarized themselves with the equipment of the VR system. They simultaneously were informed in detail about the procedure, and how to use the equipment and were encouraged to ask questions. There were given 5 min to familiarize themselves with the virtual environment and understand how to use the equipment. Then, they completed the self-efficacy expectations scale [40,41] and underwent Stroop Test [42].

They were encouraged to choose the landscape (forest, beach, snowy landscape) and the duration of their cycling performance in the VR environment. They could cycle from 5 to 30 min and were asked to cycle at a certain speed between 15–20 km/h. During their performance, they were informed on their screen about their speed, covered distance and time remaining, and could select the music they were listening to from a list of songs.

When they completed the cycling session, they underwent the Stroop test [42] and completed the self-efficacy expectations scale [40,41], a questionnaire assessing their attitudes and intention for future use [43] and their interest/enjoyment (intrinsic Motivation Inventory) [44] toward the VR exercise system. In the end, they answered a semi-structured interview with questions about their experience. The duration of the process was approximately 1 h (Table 2).

### 2.6. Measures

All participants first completed a questionnaire collecting data for their age, gender, educational level, years of substance use, days of treatment, participation in an exercise program, type of exercise, days per week, and minutes, for the TCU Drug Screen 5 according to Knight, Simpson, and Hiller, 2002 [38], IPAQ questionnaire according to Craig et al., 2003 [39]. Pre- and post-VR exercise they completed the Self-Efficacy Expectations Scale according to Bandura, 2006 [40] and Theodorakis, 1996 [41] and the Stroop Test consisting of 3 tabs (naming, reading, and interference) according to Stroop, 1935 [45].

Self-efficacy expectations were evaluated by asking subjects to rate the strength and magnitude of their self-efficacy expectations for ten performance levels from 10 to 30 min. The format used is comparable to that of Bandura [40] recommendation (e.g., “I can perform this test by 10 min”, Yes/No) and “How certain you are?” answered on a 10-point scale anchored by “certain” (10) and “uncertain” (1). The strength of perceived self-efficacy was the sum of the certainty scores for the ten levels of performance. Cronbach’s alpha for the scale was 0.88.

After their performance, they completed questionnaires about attitudes [43] toward the VR exercise and were assessed with 5 pairs of opposite words, e.g., “I find VR exercise…”—“good-bad”, “healthy-unhealthy”, etc., with scores on a differential scale of 7 points. The scoring scale ranged from 1 to 7 with higher scores indicating more positive attitudes. Intention for Future Use (IFU) of the VR system was assessed with 3 questions, e.g., “I intend to use the VR exercise system when available” with responses from “Yes sure” to “Not at all”. The scoring scale ranged from 1 to 5 with higher scores indicating greater intention for future use.

Interest/enjoyment was assessed with the relative items of the Intrinsic Motivation Inventory (IMI) [44] with 6 items, e.g., “I liked very much the VR exercise” with responses on a 1–5 Likert scale from “Not sure” to “Very sure”. The scoring scale ranged from 1 to 5 with higher scores indicating high interest/enjoyment. And at the end, they responded to a semi-structured interview consisting of 14 questions about the participants’ experience with the VR system. They were asked to describe why they would like to use the VR system to exercise, usability or utilization was assessed with 4 questions, e.g., “What difficulties did you encounter while exercising with the VR system?”, usability or learning was assessed with one question, e.g., “Did it take long to figure out how the VR system works?”, usability or pleasantness was assessed with 2 questions, e.g., “What did you like the most and what did you like least about the VR system?”, sense of presence or engagement was assessed with 2 questions, e.g., “Were you easily distracted while exercising with the VR system?”, sense of presence or realism was assessed with one question, e.g., “How did you find the environment? Realistic or artificial?” and tolerability with 3 questions, e.g., “Did you feel bad at any moment when exercising with the VR system?” (Supplementary Materials).

### 2.7. Statistical Analysis

All data are presented as means (M), Standard Deviation (SD), range, and percentages (%). Descriptive statistics, scatter plots, and frequencies were used for demographic characteristics, the VR system questions, Stroop test scores, psychological factors, and VR performance. The Kolmogorov–Smirnov Test was used to assess the normality of the distribution. Correlation among variables was assessed using the Pearson coefficient and Cronbach’s α reliability analysis was performed for all questionnaires. Paired samples *t*-test was used to identify self-efficacy expectations differences pre- and post-VR exercise. Repeated measures ANOVA analysis was used to test the effect of days of treatment on the Stroop performance. Qualitative data from the semi-structured interview were analyzed using thematic analysis, providing valuable insights into attitudes and beliefs through the identification of recurring patterns in ideas and responses [46]. All statistical analyses were performed with IBM SPSS (Statistics Version 29) and the level of significance was set at *p* < 0.05. The analysis was not pre-registered and the results should be considered exploratory.

## 3. Results

### 3.1. Pre- and Post-VR Exercise Measures

#### 3.1.1. Stroop Test

Participants experienced a statistically significant reduction in completion time for the color (Naming), word (Reading), and word-color (Interference) tasks of the Stroop test following the VR exercise. Additionally, there was a decrease in the number of errors made, although this difference did not reach statistical significance. Table 3 presents the means, standard deviations (SD), and levels of significance for all 3 tabs of the Stroop test. Furthermore, a moderate negative correlation (r = −0.45, *p* < 0.05) was observed between the time taken to complete the reading task before the VR exercise and the duration of the treatment days (M = 44.94, SD = 5.44). The Repeated Measures ANOVA analysis, including treatment days as a covariate, revealed non-significant effects across all tabs of the Stroop test. For Table 1 (naming), F (1,18) = 0.10, *p* = 0.756; Table 2 (reading), F (1,18) = 1.982, *p* = 0.176; and Table 3 (interference), F (1,18) = 0.09, *p* = 0.767.

#### 3.1.2. Self-Efficacy Expectations

A paired-samples *t*-test was conducted to compare the self-efficacy expectations pre- and post-VR exercise. There was a statistically significant improvement in self-efficacy expectations after the VR experience compared with self-efficacy expectations before the VR experience. Table 3 presents the means, standard deviations (SD), *p* value, df.

### 3.2. Post-VR Exercise Measures

#### 3.2.1. Attitudes, Intention for Future Use, Interest/Enjoyment

The participants expressed high levels of intention for future use (M = 4.5, SD = 0.6, Cronbach α 0.85), interest/enjoyment (M = 4.4 SD = 0.6, Cronbach α 0.89), and positive attitudes towards the VR exercise system (M = 6.6, SD = 0.4, Cronbach α 0.79).

#### 3.2.2. VR System Experience

**Figure 3:**
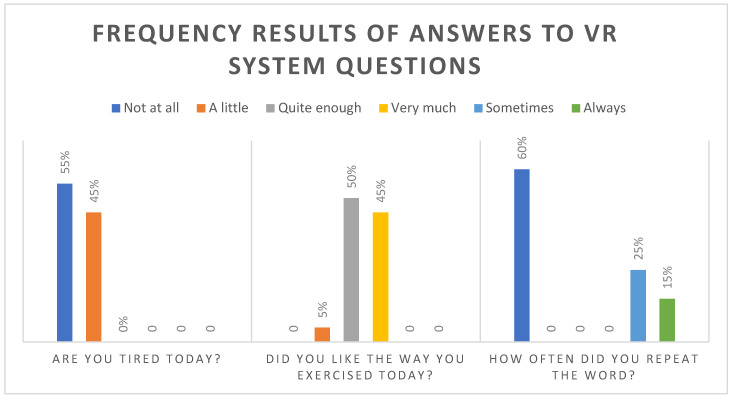
Frequency of answers to VR system questions.

Following the completion of the cycling program within the VR system, participants were asked to respond to three questions evaluating their VR cycling experience. The responses indicated that the majority of participants did not feel tired from the cycling activity and expressed a positive liking towards it. Figure 3 presents the results of these questions.

#### 3.2.3. VR Performance

In the self-selected duration of the VR exercise, 25% of the participants chose to practice for 15 min, 45% for 20 min, and 30% for 30 min. All participants achieved their exercise goal and we did not have dropouts. The Mean and SD of their cycling time was 21.8 ± 5.9 (Range 15.0–30.0). Their average speed was 19.05 with the guidance to cycle between 15 and 20 km/h (M = 19.1, SD = 0.7, Range 17.14–20.21) and an average of covered distance of 6.19 km (M = 6.2, SD = 1.7, Range 4.0–8.9).

**Figure 4:**
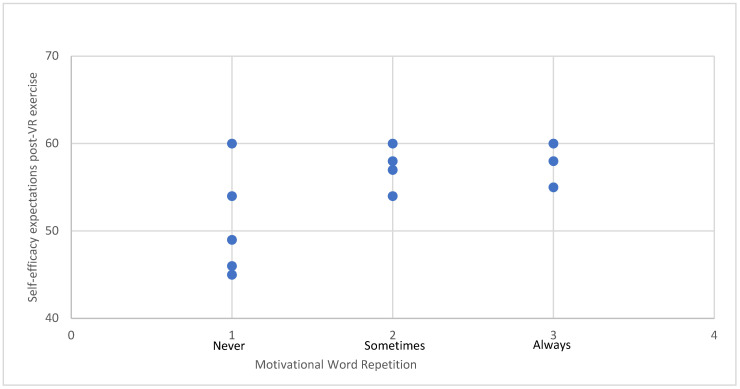
Scatter plot: a relationship between self-efficacy and motivational word usage.

The scatter plot (Figure 4) observation indicates that those who used motivational words had higher scores in self-efficacy expectations. Nevertheless, despite this observation, both regression and correlation analyses failed to yield statistically significant findings. Specifically, the regression analysis indicated a multiple R of 0.021, which did not significantly deviate from zero (F (1,18) = 0.387, *p* = 0.542). Similarly, Spearman’s correlation analysis also did not reveal a statistically significant correlation between the variables (*p* = 0.715).

### 3.3. Technical Functionality, User Experience, and Emotional Responses toward the VR Exercise System

A thematic analysis was conducted to analyze the qualitative data obtained from the semi-structured interviews, and the findings are presented in Table 4. According to the participants’ reports, they did not encounter any technical issues and did not require additional time to understand the VR exercise system. The majority of participants expressed satisfaction with the VR system equipment, including the head-mounted display and joystick, although a few mentioned that the mask felt slightly heavy or tight. None of the participants experienced negative emotions, discomfort, or unusual thoughts during the cycling activity, except for one individual who initially felt some dizziness for a few seconds at the beginning of the VR experience. All participants expressed a desire to continue using the VR system for exercise, emphasizing that they found it enjoyable, relaxing, motivating, interesting, challenging, and capable of evoking feelings of happiness. They also mentioned that the system provided a means of escaping from reality and was easy to use.

## 4. Discussion

In our feasibility study, our objective was to investigate the self-efficacy expectations and attentional control differences of individuals in SUD treatment pre- and post-exercise with the VR system and to investigate their attitudes, intentions for future use, and interest/enjoyment about this type of exercise, immediately after the exercise session. The results of this study offer insights into the impacts of an VR exercise system on exercise self-efficacy expectations and performance in the Stroop test. Additionally, the results indicated positive outcomes and acceptance of the VR exercise system for people under SUD therapy, thus suggesting the feasibility of this approach.

### 4.1. The Effects of VR Exercise on Self-Efficacy Expectations of Individuals in SUD Treatment

In our study, we evaluated self-efficacy expectations before and after the VR exercise. Consistent with Bandura’s definition [18], these expectations reflect an individual’s belief in their capability to complete tasks essential for desired outcomes in specific situations. Our findings revealed a significant improvement in self-efficacy expectations following the VR experience. Panagiotounis et al., 2020 [47] assessed self-efficacy in individuals in the treatment of SUD. According to this study, the intervention lasted 5 days and was an adventure-based therapy for the promotion of participants’ therapeutic change involving physical activity. The results have shown that the participants’ self-efficacy and self-esteem were positively affected [47]. Our findings did not unveil any significant relationship between self-efficacy and the repetition of motivational words; rather, this observation was discerned solely from the scatter plot. It is possible that the limited sample size may account for this outcome. According to our results, there were statistically significant differences in self-efficacy expectations, that can be positively influenced by one bout of exercise. This can be elucidated by emphasizing that, as previously noted, self-efficacy expectations are more precise, and engaging in exercise provides immediate successful experiences that strengthen individuals’ confidence in their ability to participate in physical activity [18]. From our perspective, VR exercise shows promise as a tool for indirectly enhancing self-efficacy after a few times of exercising as we found improvements in self-efficacy expectations scores in acute exercise.

### 4.2. The Effects of VR Exercise on the Attentional Control of Individuals in SUD Treatment

According to our results, there was a statistically significant reduction in reaction time in the Stroop test after the VR exercise. It was observed that individuals with important positions with many demands and responsibilities for the community, such as drivers and coordinators noted better scores in all tabs of the Stroop test compared with members who were less active in their community. Nevertheless, covariate analysis did not find a significant effect. Our results are consistent with those of Smith et al., 2021 [48], supporting the observation that participants who engaged in a VR exercise program as part of their treatment showed significant improvements in Stroop test performance after VR exercise [49]. Based on our findings, it is plausible to suggest that VR exercise may contribute to enhancing cognitive restoration during therapy. Likewise, previous research has indicated that individuals with SUD demonstrated decreased reaction time in the Stroop test following an acute bout of Tai Chi or high-intensity interval training, with the latter demonstrating superior improvement [50]. To investigate the potential influence of a practice effect on the substantial improvement of reaction time observed in the Stroop test, our Stroop test effect sizes (Cohen’s d) were compared with those of Zimmer et al., 2016 [49], who utilized the same test in a pre-post design with a control group, notable differences emerged. The comparison of our Stroop test effect sizes with those of Zimmer et al., 2016 [49] provides valuable insights into the potential impact of exercise intervention on cognitive function. Despite the acknowledged vulnerability of the Stroop test to practice effects, our study revealed significant differences in effect sizes, particularly in the naming and interference tabs, indicating a potential contribution from acute exercise. The higher Cohen’s d values observed in these tabs compared to the control group of Zimmer et al., 2016 [49] suggest temporary cognitive changes that extend beyond mere familiarity with the test. We also found significant relations between days in the treatment and the Stroop test pre-exercise score in the second tab of the Stroop test. There was a moderate negative relation between these variables and this can be a clue that maybe the days spent in abstinence from drugs may influence positively the cognitive function. Nevertheless, repeated measures ANOVA with covariate analysis on the relationship before and after VR training Stroop test scores showed a non-significant effect of days in treatment. Research has consistently shown that longer durations of abstinence from drugs are associated with improved cognitive function, including attention, working memory, and executive control [51,52].

In our study, all participants were poly-users, but the vast majority of them reported that heroin was the substance that was the most difficult to abstain process. Heroin addiction adversely impacts impulse control, attention, and flexibility. Additionally, heroin craving levels can predict how quickly someone reacts to heroin-related cues [53]. However, because the sample size was small and all participants used multiple substances, no comparative analyses were conducted to assess the impact of specific drugs on their Stroop test performance.

### 4.3. The Attitudes, Intention for Future Use, and Interest/Enjoyment of Individuals in SUD Treatment toward VR Exercise

According to the results, most participants showed increased levels of interest/enjoyment, intention for future use, and positive attitudes toward the VR exercise system indicating positive perceptions and high motivation. Interest/enjoyment is a dimension of intrinsic motivation and the high scores indicate positive motivation. These results suggest that the participants after using the VR exercise system, had a favorable attitude toward the VR exercise system, intended to use it in the future, and expressed strong interest and enjoyment during the VR cycling experience. These results align with participants’ feedback regarding their virtual experience from the interview. Notably, there were no reports of adverse effects such as nausea or dizziness, and overall comments on the VR exercise application were highly positive. However, several participants mentioned that they found the landscape to be somewhat monotonous. Although this feedback is valuable, it should be considered for future studies utilizing the VRADA application. Furthermore, the feedback gathered from questions presented on the screen following the cycling performance was overwhelmingly positive. The majority of participants expressed satisfaction with their exercise experience and reported no feelings of fatigue. However, there were fewer positive responses regarding the word repetition task, indicating that some participants found this aspect less enjoyable or engaging.

In our study, participants chose their preferred landscape (forest, beach, snowy landscape) and cycling duration (5 to 30 min) in the VR environment, receiving real-time feedback on speed, distance, and remaining time, while also having the option to select music from a list of songs. Research consistently demonstrates that incorporating choices into individuals’ exercise routines significantly enhances their experience and motivation. By allowing participants to select key elements such as exercise duration, preferred music, and the surrounding landscape, exercise programs become powerful tools for providing autonomy. This practice aligns with motivational techniques rooted in self-determination theory, fostering intrinsic motivation and a greater sense of ownership over one’s fitness journey [54]. The ability to tailor exercise programs to individual preferences not only makes them more personalized but also increases engagement and enjoyment. Ultimately, these autonomy-supportive strategies contribute to a positive and individualized exercise experience, promoting sustained adherence and overall well-being.

VR exercise arose as a promising tool for individuals in SUD treatment, as it offers a unique and immersive approach to physical activity. Studies assessing the effects of VR exercise on individuals in SUD treatment have found positive outcomes in terms of interest/enjoyment, attitudes towards exercise, self-efficacy, and cognitive function measured through the Stroop test. For instance, a study by Nesbitt et al., 2020 [55] investigated the use of VR exercise among individuals in SUD treatment and found that participants reported high levels of interest and enjoyment during VR exercise sessions [54]. Another study demonstrated that VR exercise interventions were associated with improved attitudes towards exercise and increased self-efficacy among individuals in SUD treatment [56]. Furthermore, other researchers investigated the effects of VR exercise on cognitive function using the Stroop test in individuals with SUD and found significant improvements in attention and inhibitory control following VR exercise sessions [57]. According to Worley (2019), VR can be a promising tool to help individuals under SUD treatment to manage cravings and pain, reduce psychological issues, learn life skills and exercise [58]. However, there are not many studies comparing VR exercise programs with traditional exercise. Qian et al. 2020, highlight that engaging in VR exercise can offer significant benefits to physical health, mental well-being, and the recovery process across various populations, providing distinct advantages compared to traditional exercise methods [30]. VR is, in general, increasingly recognized as a valuable tool for exercise therapy across various disorders requiring rehabilitation. It addresses the limitations of traditional exercise methods by offering a more engaging and effective approach. VR programs have demonstrated superior outcomes compared to conventional exercise therapies, making them a preferred choice in many cases. These programs are versatile and can be tailored for numerous therapeutic purposes, including pain management, enhancing functional abilities, increasing range of motion, promoting muscular strength, and boosting motivation. Consequently, VR represents a promising advancement in the field of exercise therapy, providing comprehensive benefits for various populations including individuals in SUD treatment [59]. Therefore, exercise in virtual environments offers all the benefits of traditional exercise, such as improved physical health and well-being, while also enhancing enjoyment and motivation.

### 4.4. Practical Implications

The findings of our study have several practical implications for the integration of VR exercise systems in SUD treatment programs. The significant improvements in self-efficacy expectations and attentional control observed after a single session of VR cycling suggest that this type of intervention could be a valuable addition to traditional SUD treatment modalities. Firstly, the high levels of interest, enjoyment, and positive attitudes towards the VR exercise system indicate that such technology can make exercise more appealing to individuals undergoing SUD treatment. By increasing motivation and engagement through enjoyable and immersive experiences, VR exercise systems can help in maintaining regular physical activity, which is crucial for the overall well-being and recovery process of individuals with SUD [60]. Also, the observed improvements in attentional control, as evidenced by the Stroop test results, highlight the potential cognitive benefits of incorporating VR exercise into SUD treatment. Enhanced attentional control can contribute to better decision-making and impulse control, which are critical components in preventing relapse and promoting sustained recovery [61]. The significant increase in self-efficacy expectations post-exercise suggests that VR cycling can boost confidence in one’s ability to engage in and benefit from physical activity. Higher self-efficacy is associated with greater persistence in the face of challenges, which can be particularly beneficial for individuals recovering from SUD [22]. The ability to engage in VR exercise sessions at self-selected durations and intensities provides flexibility, making it easier to tailor interventions to individual preferences and needs. This personalized approach can enhance adherence to exercise programs and ensure that participants are neither overwhelmed nor under-challenged. Hence, by participating in exercise programs, these individuals can achieve better physical and mental health outcomes and more positive substance use outcomes [62]. Implementing exercise through a VR environment can further enhance motivation and engagement and offer access to safe and appealing exercise environments leading to increased self-efficacy and improved cognitive functions. These improvements are inversely related to relapse and craving symptoms, making exercise an essential component of effective SUD treatment strategies. A VR exercise system can be an effective tool to increase their participation to exercise programs, maintain a regular exercise routine and provide substantial benefits, reducing the risk of dropout and enhancing recovery outcomes.

### 4.5. Strengths & Limitations

This study contributes significantly to the emerging field of VR exercise interventions for individuals undergoing SUD treatment. One of its primary strengths lies in its holistic approach, exploring not only the cognitive benefits but also participants’ attitudes, intentions, and interest/enjoyment related to VR exercise. The utilization of both quantitative measures, such as the Stroop test for attentional control, and qualitative data offers a comprehensive understanding of the intervention’s impact.

Despite its strengths, this study has several limitations that warrant consideration. The findings may not be widely applicable to a larger population due to the relatively small sample size. The study’s focused exploration within a specific population undergoing SUD treatment, coupled with resource constraints for intensive assessments, necessitated a smaller sample size of 20 participants. Although limited in generalizability, this approach allowed for in-depth insights and laid the groundwork for future, more extensive investigations. Recruiting participants from this population posed a considerable challenge. Despite accessing the largest treatment center in the country, achieving a more homogeneous sample was unattainable. In general, recruiting participants from this population is challenging because of their uncertain retention in the SUD treatment program, limited availability of participants, the need for special consent due to the sensitive nature, and the variability in participants’ drug use history and treatment duration. The heterogeneity of the sample should be considered when interpreting the results, as they may influence the findings in ways not entirely accounted for by our analyses. Future research should aim to recruit a larger and more homogeneous sample to validate and extend the findings of this study. Despite these limitations, the study provides valuable insights into the experiences of individuals undergoing treatment for SUD, highlighting the need for tailored treatment approaches that consider individual variability. The absence of a control group and random assignment is acknowledged, posing challenges in establishing causal relationships. The quasi-experimental design, while providing initial insights, necessitates caution in generalizing findings to long-term effects, highlighting the need for future studies with extended interventions and control conditions. According to Edwards et al., 1996 [63], it is possible that the practice effect from the Stroop test may have influenced the positive results regarding attention. Therefore, because our study design did not include a control group, we might have had the effect of practice when we compared the before–after Stroop test scores. Nevertheless, previous studies examining the effect of exercise on Stroop performance, indicated that an acute bout of exercise led to improved scores in the Stroop test, compared with a control group [64]. According to our findings the effect size of the Stroop task in our study was higher compared to the control group of Zimmer et al., 2016 [49]. These findings underscore the nuanced effects of exercise on cognitive performance and underscore the necessity for additional research to elucidate the mechanisms underlying these effects. Additionally, in this study, we observed that individuals with important positions and numerous demands and responsibilities in the community, such as drivers and coordinators, achieved better scores in all tabs of the Stroop test compared to members who were less active in their community. This observation underscores the importance of considering such factors in future studies on this topic. In conclusion, there is a need for further research involving extended exercise interventions utilizing VR environments, larger sample sizes, randomized controlled trials, and prolonged follow-up periods. These endeavors will serve to validate and expand upon the current findings, shedding light on whether such exercise modalities could contribute to reduce drug use and cravings, or to enhance recovery self-efficacy.

## 5. Conclusions

In conclusion, VR exercise can be a useful and promising tool to be used supplementally for SUD treatment. A short bout of self-selected exercise in a virtual environment can lead to increased scores in cognitive factors, enhance self-efficacy expectations levels, and can be a motivating way to improve physical activity participation levels of individuals with SUD. Further research in this field could explore the long-term effects and sustained benefits of VR exercise interventions for individuals undergoing SUD treatment. Additionally, investigations employing randomized controlled trials with larger and more diverse samples, extended intervention periods, and robust control conditions would contribute to establishing the efficacy and generalizability of such interventions, providing valuable insights for clinical applications.

## Figures and Tables

**Table 1:** Demographic characteristics.

N = 20		Mean	SD	Minimum	Maximum
Age	Years	37.75	8.33	24	54
Sex	Male, %	90	-	-	-
Educational level	Primary, % Secondary, %	85% secondary 10% primary	-	-	-
Exercise during treatment	Yes, %	50	-	-	-
Current engagement in SUDs treatment	Days	138.8	106.25	6	341
Previous attempts	Times	1.80	1.642	0	4 and more
	Frequency (%)	Never 35% 1 time 15% 2 times 5% 3 times 25% 4 plus 20%			
Drug use	Years	19.4	9.88	7	37

**Table 2:** Experimental procedure.

Pre-VR Exercise		Post-VR Exercise
Consent form demographics TCU drug screening DASS-21 IPAQ Familiarization (5 min) Self-efficacy expectations Stroop test	**Self-selected duration VR exercise**	Stroop test Self-efficacy expectations Attitudes Intention for future use Intrinsic motivation inventory Semi-structured interview

Note: VR = Virtual Reality, TCU = Texas Christian University Questionnaire, IPAQ = International Physical Activity Questionnaire, DASS-21 = Depression Anxiety Stress Scales—21 items.

**Table 3:** Stroop test reaction time and mistakes and self-efficacy expectations. All data are presented as means and Standard Deviation (SD).

Stroop Test		Pre VR	Post VR	*p* Value, df	Cohen’s d
**Naming**	Reaction time	60.9 ± 13.1	51.9 ± 7.6	t(19) = 4.178, *p* < 0.001	0.84
	Mistakes	1.1 ± 1.2	0.6 ± 0.9	t(19) = 1.308, *p* = 0.21	
**Reading**	Reaction time	41.9 ± 5.4	40.2 ± 6.2	t(19) = 3.202, *p* < 0.005	0.29
	Mistakes	0.1 ± 0.2	0.1 ± 0.2	t(19) = 0.152, *p* = 1.0	
**Interference**	Reaction time	94.8 ± 22.9	78.0 ± 14.1	t(19) = 6.242, *p* < 0.001	0.88
	Mistakes	4.4 ± 4.1	2.6 ± 0.7	t(19) = 1.966, *p* = 0.06.	
**Self-efficacy expectations**		49.8 ± 13.19	56.8 ± 4.87	t(19) = −2.78, *p* < 0.01	-

**Table 4:** Τhematic analysis of the semi-structured interview table.

Main Theme	Subthemes	N	(%)
**Reasons to use VRADA**	*Why would you use VRADA?*		
	It is pleasant	14	70%
	It is relaxing	8	40%
	It is motivating	3	15%
**Usability or utilization**	*General difficulties*		
	None	16	80%
	Difficulty adjusting to the speed limits	3	15%
	*Technical issues*		
	None	20	100%
	*Joystick use difficulty*		
	No	20	100%
	*VR mask use difficulties*		
	None	16	80%
	It was a little heavy	2	10%
	It was a little tight	2	10%
**Usability or learning**	*Need more time to understand the system*		
	No	20	100%
**Usability or pleasantness**	*Most enjoyable parts*		
	The VR environment (landscape)	15	75%
	Music	3	15%
	Peacefulness and relaxation	3	15%
	*Least enjoyable parts*		
	Repeated virtual parts, monotonous	9	45%
	Graphics	8	40%
	Music	5	25%
	*Feel uncomfortable*		
	No	20	100%
**Sense of presence or engagement**	*Distraction of attention*		
	No	19	95%
	Yes	1	5%
	*Duration of the experience*		
	It could be more	10	50%
	It was enough	7	35%
	It could be more but I did not have time because of our demanding schedule	3	15%
**Sense of presence or realism**	*VR environment was realistic or artificial*		
	Artificial	20	100%
**Tolerability**	*Feel bad during training*		
	Νο	20	100%
	*Thinking of weird things*		
	Νο	20	100%
	*Nausea, dizziness or other physical symptoms*		
	Νο	19	95%
	A little for the first seconds	1	5%

## Data Availability

The data presented in this study are available on request from the corresponding author due to privacy reasons.

## Supplementary Materials

The following supporting information can be downloaded at: https://www.mdpi.com/article/10.3390/brainsci14070724/s1, Interview Guide (semi-Structured).
