# Supplementary material for: Exploring the Acute Effects of Immersive Virtual Reality Biking on Self-Efficacy and Attention of Individuals in the Treatment of Substance Use Disorders: A Feasibility Study
Source: Brain Sci. 2024 Jul 18;14(7):724. doi: 10.3390/brainsci14070724 (PMC11274936; doi:10.3390/brainsci14070724)
Supplement: Supplementary file 1 [file brainsci-14-00724-s001.zip › brainsci-3091392-supplementary.pdf]

Supplementary Materials

Interview Guide (semi-structured)

1. Why would you want to use the Virtual Reality system to exercise?
2. What difficulties did you encounter while exercising with the Virtual Reality system?
3. Were there any technical issues while exercising with the Virtual Reality system?
4. Did it take long to figure out how the Virtual Reality system works? What took most time to figure out? Was the trial sufficient to comprehend the Virtual Reality system?
5. Was the use of the joystick difficult/complicated (e.g., buttons)? What aspect of using the joystick gave you most trouble?
6. What did you like most and what did you like least about the Virtual Reality system?
7. Was there any point at which the Virtual Reality system made you feel uncomfortable?
8. What problems (if any) did you have with the Virtual Reality mask during the stationary bike exercise?
9. What do you think about the duration of the experience?
10. Were you easily distracted while exercising with the Virtual Reality system? At what point did you get distracted?
11. How did you find the environment? Was it realistic or too artificial?
12. Did you feel bad at any moment when exercising with the Virtual Reality system?
13. Was there any point during the Virtual Reality system exercise that made you think of weird things? If yes, what was that?
14. Did you experience any nausea, dizziness or other physical symptoms when exercising with the Virtual Reality system?
